# Artificial intelligence in the transition of allergy: a valuable tool from childhood to adulthood

**DOI:** 10.3389/fmed.2024.1469161

**Published:** 2024-08-15

**Authors:** Cristiana Indolfi, Angela Klain, Giulio Dinardo, Fabio Decimo, Michele Miraglia del Giudice

**Affiliations:** Department of Woman, Child and General and Specialized Surgery, University of Campania 'Luigi Vanvitelli', Naples, Italy

**Keywords:** artificial intelligence, transition, children, adults, asthma, allergy

## 1 Introduction

Artificial intelligence (AI) is having a revolutionary effect in several industries, including healthcare. Nowadays AI plays an increasingly significant role in managing chronic diseases. A study published in *The Lancet Digital Health* highlighted the effectiveness of AI-driven virtual health assistants in improving medication adherence among patients with chronic illnesses (1). The study by Downing et al. (2), demonstrated that Livongo's AI-driven approach significantly improved glycemic control and reduced the incidence of diabetes-related complications. A recent study published in *Nature* demonstrated that AI could predict adverse cardiovascular events by analyzing electronic health records, helping to tailor preventive measures more effectively than traditional approaches (3).

AI has the potential to completely transform our understanding, diagnosis, treatment, and management of allergic diseases including asthma (4–6). AI has already been demonstrated to be useful in allergic diseases in children. In the study by Smith et al., AI was used to analyze electronic health records data to diagnose asthma from 500 pediatric patients who presented with respiratory symptoms. The results were compared with traditional diagnostic methods. The AI tool demonstrated an accuracy of 92%, sensitivity of 89%, and specificity of 94% in diagnosing asthma, outperforming traditional methods which had an accuracy of 85%, sensitivity of 82%, and specificity of 88% (7).

AI, used as machine learning, demonstrated to be superior to traditional diagnostics also in allergic rhinitis, chronic cough, and respiratory infections (8–10).

Conversely, as of now, there are no studies on the effectiveness of AI in managing the transition from pediatric to adult stages in allergic diseases. The transition is one of the biggest obstacles for people with asthma and allergies, as well as their families. The European Academy of Allergy and Clinical Immunology (EAACI) has developed evidence-based guidelines for healthcare professionals to support the transitional care of adolescents and young adults with allergic diseases and/or asthma. These recommendations advocate for early transition initiation (11–13 years), a structured multidisciplinary approach, patient education, medication simplification, psychological support, and involving peers and family in self-management (11).

Patients with allergies face several unique obstacles while moving from pediatric to adult treatment, which can negatively affect their health and quality of life. Adherence to drug therapy is one of the main issues. Due to forgetfulness, a lack of awareness of the significance of adherence, or dealing with unpleasant side effects, adolescents and young adults sometimes find it difficult to maintain regular adherence to prescribed drugs. This inconsistency might worsen symptoms and raise the likelihood of serious allergic responses. Another key problem is self-management. As patients reach maturity, they are expected to take on increasing responsibility for controlling their allergies. This includes recognizing symptoms, avoiding triggers, and understanding how to utilize emergency medications like epinephrine auto-injectors. Many young individuals may be unprepared for this additional responsibility, which can lead to worry and failures in good management. Communication breakdowns between healthcare professionals exacerbate the shift. Effective transition necessitates smooth communication between pediatric and adult healthcare practitioners, which is sometimes hampered by disparate medical record systems and a lack of standardized processes for transferring care. Consequently, critical information about the patient's allergy history and management plan may not be adequately communicated, leading to fragmented care and potential health risks.

Moving from the care of a pediatric specialist who has followed the patient for years, providing regular follow-ups and understanding both clinical and personal details (such as treatment difficulties, friendships and school issues), often makes it difficult to detach from that trusted figure (12). The family and the patient frequently request continued care from the pediatrician even into adulthood because they trust them and are concerned about relying on a new professional unfamiliar with the patient's medical history. Pediatricians who have cared for their patients since childhood possess valuable insights into their medical history, treatment plans, and individual needs. Transitioning to a new healthcare provider can be challenging, as it involves navigating unfamiliar territory and establishing trust with a new professional figure. It is not merely a matter of transferring medical records. It requires establishing a new therapeutic relationship built on trust and comprehensive knowledge of the patient's clinical history, especially if the patient has a complex allergy history. For example, patients with multiple food allergies undergoing years-long desensitization processes or those with asthma or atopic dermatitis receiving biologic therapy who require specialized care and monitoring. The transition can also pose a real problem for patients with co-morbidities. Continuity of care during this period is crucial for maintaining the patient's wellbeing and ensuring their medical needs are adequately addressed.

The purpose of this article is to explore the potential of AI in addressing these specific challenges faced by allergic and asthmatic patients. We will examine how AI can improve adherence to therapy, enhance self-management practices, and bridge communication gaps between pediatric and adult care providers, ultimately aiming to improve the overall transition experience for allergic patients.

## 2 How AI can aid in the transition of allergic diseases?

AI can be particularly beneficial during the transition in two critical phases: (a) assisting patients in taking responsibility for independently managing their disease and (b) facilitating the transfer of information from the pediatric allergist to the allergy specialist.

### 2.1 Assisting patients in taking responsibility

Adolescents transitioning to adulthood face the challenge of moving from a parent-guided healthcare regimen to independently managing their health. The use of AI-driven mobile apps can offer reminders for medication adherence, monitor symptoms, and suggest lifestyle adjustments based on real-time data. These apps can educate patients about their condition, treatment plans, and the importance of adhering to prescribed therapies. Interactive features, such as virtual health assistants, can answer questions, provide motivational support, and offer feedback on the patient's self-management practices. AI can also be useful in managing the switching of the device used for inhalation therapy, from the spacer to the diskus for asthma.

Adolescents and adults need to learn to carry and self-administer adrenaline effectively and safely. AI can assist patients in learning and independently managing the administration of intramuscular adrenaline. Additionally, AI can analyze patterns in the patient's health data to predict potential exacerbations or allergic reactions, allowing patients to take preemptive action. For example, if an AI system detects an increase in environmental allergens that typically trigger a patient's asthma, it can alert the patient to take preventive measures, such as using inhalers or avoiding certain activities. Despite being required by law, some restaurants fail to show the allergy list or don't consider the possibility of contamination. In the near future, AI could be able to identify and indicate the existence of allergens in a meal by detecting traces.

Moreover, AI can facilitate telemedicine consultations, where patients can report their symptoms and receive guidance without needing to visit the doctor's office. Simulation scenarios, supported by virtual reality, can be valuable in enhancing a patient's autonomy in treating and managing their condition. This empowers patients to handle minor issues independently while having support readily available if needed.

### 2.2 Facilitating the transfer of information

AI technology offers an opportunity to streamline the transition process. By analyzing vast amounts of data, including electronic health records, laboratory results, and treatment protocols, AI algorithms can provide comprehensive insights to improve continuity of care. AI can be instrumental in building a patient's report, encompassing history, recent therapies, comorbidities, and treatment advances. The assignment of disease risk (such as asthma exacerbation or anaphylaxis) and the construction of predictive models can further support clinicians in making informed decisions during the transition period. By extracting insights from electronic health records, AI systems can ensure that the new healthcare provider is well-informed about the patient's condition and previous treatments.

## 3 Limitations of AI in the transition of allergy care

While AI offers significant potential benefits in managing the transition of allergy care from childhood to adulthood, several limitations must be considered. First, the accuracy and effectiveness of AI systems heavily rely on the quality and comprehensiveness of the data they are trained on. Incomplete or biased data can lead to incorrect predictions and recommendations, potentially jeopardizing patient safety. The effectiveness of AI in healthcare heavily relies on the quality and diversity of the datasets used for training algorithms. High-quality, diverse datasets are crucial to avoid biased or inaccurate predictions. Bias in AI can arise from unrepresentative data that fails to include various demographic groups, leading to disparities in healthcare outcomes. For instance, an AI system trained predominantly on data from a specific ethnic group may not perform well for individuals from different backgrounds. A recent study highlighted how biased datasets can lead to significant disparities in healthcare delivery and outcomes (13). Ensuring data diversity and quality helps create more equitable AI applications in healthcare, providing accurate predictions and treatment recommendations for all patient groups. The deployment of AI in healthcare also brings to the forefront significant ethical and privacy issues. Healthcare providers must ensure that AI applications comply with stringent regulatory standards to protect patient information. Robust data protection measures are essential to safeguard patient information and maintain confidentiality. This involves implementing stringent security protocols and ensuring compliance with regulations such as the General Data Protection Regulation (GDPR) (14). Additionally, transparency in AI algorithms is critical to building trust among patients and healthcare providers. Transparent algorithms allow stakeholders to understand how decisions are made, facilitating accountability and ethical use (15).

While AI offers substantial benefits, it is crucial to recognize that it cannot replace the expertise and empathy of healthcare professionals. AI should be viewed as a complementary tool that enhances the capabilities of healthcare providers rather than replacing them. The human touch, characterized by empathy, ethical judgment, and nuanced understanding of patient needs, remains irreplaceable. For example, while AI can analyze data and suggest diagnoses, the interpretation of these suggestions and the delivery of care require the human element to ensure compassionate and personalized patient care (16).

Moreover, there is a need for extensive training for both healthcare professionals and patients to effectively use AI-driven technologies. Resistance to change and a lack of trust in AI systems among patients and clinicians can also hinder widespread adoption. Ensuring a balanced approach that leverages AI's strengths while acknowledging its limitations is crucial for the successful integration of AI in transitional allergy care.

**Figure 1:**
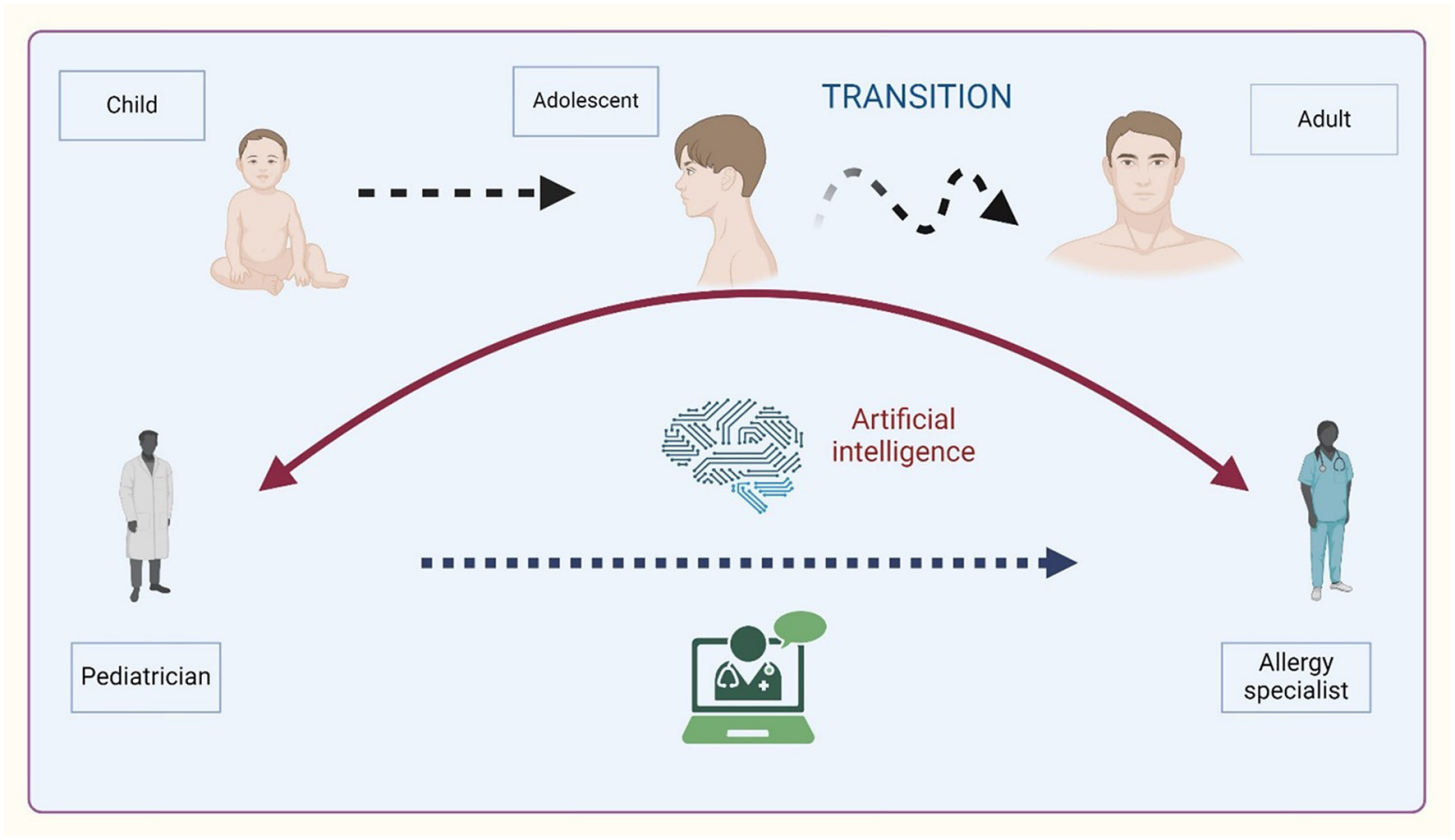
Overview of asthma transition from childhood to adulthood. Created with BioRender.com.

A graphical overview of asthma transition is illustrated in Figure 1.

## 4 Discussion and conclusion

Overall, AI has the potential to revolutionize the transition of patients with asthma and allergic diseases by improving personalized care, risk stratification, treatment optimization, remote monitoring, patient education, and provider collaboration. We think AI is a great tool to help doctors, who should learn how to use and manage it according to its appropriate use. Healthcare providers may improve patient transition experiences and guarantee continuity of treatment across the lifespan by leveraging AI-driven solutions.
